# CPAD, Curated Protein Aggregation Database: A Repository of Manually Curated Experimental Data on Protein and Peptide Aggregation

**DOI:** 10.1371/journal.pone.0152949

**Published:** 2016-04-04

**Authors:** A. Mary Thangakani, R. Nagarajan, Sandeep Kumar, R. Sakthivel, D. Velmurugan, M. Michael Gromiha

**Affiliations:** 1 Center for Advanced Studies in Crystallography and Biophysics and Bioinformatics Infrastructure Facility, University of Madras, Chennai, 600025, India; 2 Department of Biotechnology, Bhupat and Jyoti Metha School of Biosciences, Indian Institute of Technology Madras, Chennai, 600036, India; 3 Biotherapeutics Pharmaceutical Sciences, Pfizer Inc., 700 Chesterfield Parkway West, Chesterfield, Missouri, 63017, United States of America; University of Akron, UNITED STATES

## Abstract

Accurate distinction between peptide sequences that can form amyloid-fibrils or amorphous β-aggregates, identification of potential aggregation prone regions in proteins, and prediction of change in aggregation rate of a protein upon mutation(s) are critical to research on protein misfolding diseases, such as Alzheimer’s and Parkinson’s, as well as biotechnological production of protein based therapeutics. We have developed a Curated Protein Aggregation Database (CPAD), which has collected results from experimental studies performed by scientific community aimed at understanding protein/peptide aggregation. CPAD contains more than 2300 experimentally observed aggregation rates upon mutations in known amyloidogenic proteins. Each entry includes numerical values for the following parameters: change in rate of aggregation as measured by fluorescence intensity or turbidity, name and source of the protein, Uniprot and Protein Data Bank codes, single point as well as multiple mutations, and literature citation. The data in CPAD has been supplemented with five different types of additional information: (i) Amyloid fibril forming hexa-peptides, (ii) Amorphous β-aggregating hexa-peptides, (iii) Amyloid fibril forming peptides of different lengths, (iv) Amyloid fibril forming hexa-peptides whose crystal structures are available in the Protein Data Bank (PDB) and (v) Experimentally validated aggregation prone regions found in amyloidogenic proteins. Furthermore, CPAD is linked to other related databases and resources, such as Uniprot, Protein Data Bank, PUBMED, GAP, TANGO, WALTZ etc. We have set up a web interface with different search and display options so that users have the ability to get the data in multiple ways. CPAD is freely available at http://www.iitm.ac.in/bioinfo/CPAD/. The potential applications of CPAD have also been discussed.

## Introduction

Aggregation of proteins and peptides is a ubiquitous, yet poorly understood phenomenon in biochemistry. Aggregation of endogenous proteins causes several neurodegenerative and chronic diseases in humans and animals [1,2]. In biotechnology, aggregation remains the most common obstacle in the successful development and manufacturing of protein based drug products [3]. In addition to these, creation of protein and peptide aggregates with well-defined morphologies is of interest for development of novel nano-materials with desired mechanical characteristics [4]. Due to the multiple roles played by aggregation, it has become an active area of biophysical research in recent decades.

The mechanisms, origins and implications of protein aggregation have been extensively studied with both experimental and computational methods [5–8]. Serrano’s group derived hexa-peptide patterns susceptible to aggregation at neutral and acidic pH by examining variants of a *de novo* designed amyloid fibril forming hexa-peptide, STVIIE [6]. Eisenberg’s group [9] elucidated the molecular features of amyloid fibrils containing cross-β motifs of 5–9 residues long in atomic details by determining their three-dimensional structures *via* single crystal X-ray diffraction. Currently, several crystal structures of aggregating peptides are available in Protein Data Bank [10]. Tsolis *et al*. [7] collected the experimental observations on aggregation prone regions (APRs) on several amyloidogenic proteins. These regions are of varied lengths and most of them are in the range of 6–72 residues. Further, the influence of amino acid mutations on protein aggregation has been studied experimentally using ThT fluorescence and Congo Red stains [5,11,12]. Systematic collection and curation of accumulating experimental data is necessary to understand the mechanism(s) of protein aggregation and to develop computational algorithms.

Several computational approaches have been developed to understand the influence of amino acid properties such as hydrophobicity, β-strand propensity, charge and solubility of amyloid forming peptides for protein aggregation [13], distinguishing between amyloid fibril forming and amorphous β-aggregating peptides, and detecting aggregation protein regions (APRs) in protein sequences [8,14]. It has also been reported that hydrophobicity and aggregation propensity of amino acid residues are important for understanding change in aggregation rates of proteins upon mutation [15]. Although experimental data on aggregating peptides are reported in the literature [14–17] and available in different databases [18], there is no combined manually curated database available for the broad collection of experimental data on aggregating peptides, aggregation prone regions and aggregation rates upon mutations.

In this work, we have developed CPAD, a manually Curated Protein Aggregation Database. CPAD contains data collected from experimental studies available in the literature on aggregation. The data from these studies has been manually curated into three classes with a focus on Aggregation Rate Change upon Mutation(s) (ARCM). ARCM contains experimental data on change in rate of protein aggregation upon single point as well as multiple mutations, along with sequence, structure and literature information. Further, CPAD is supplemented with information on Aggregating Peptides (AP) and Aggregation Prone Regions (APRs). The aggregating peptides have been further divided into four categories, *viz*., amyloid fibril forming and amorphous β-aggregating hexa-peptides, amyloid fibril forming peptides of different lengths, amyloid fibril forming peptides with three-dimensional structures available in the Protein Data Bank. The class APR contains experimentally validated aggregation prone regions (APRs) in amyloidogenic proteins. We have set up a web interface for integrating all types of data and users have flexibility to download any or all data of interest.

## Contents of the Database

CPAD is an integrated database on a total of six categories of protein and peptide aggregation, which are grouped into three classes, change in protein aggregation rates upon mutations (ARCM), aggregating Peptides (AP) and aggregation prone regions in amyloidogenic proteins (APRs).

### Aggregation rate change upon mutation(s) (ARCM)

Experimental studies available in the literature show that the mutation of specific residues in proteins can increase or decrease their aggregation rates. We have searched PUBMED and other online resources to obtain the data on change in protein aggregation rates upon mutations. Each entry of the database includes the following information:

#### Sequence and structure information

Name, source, length of the protein, Uniprot code [19], PDB code [10], mutation (for example, mutation of A to V at position 6 is mentioned as A6V), secondary structure (helix, strand or coil) and accessible surface area, ASA. The secondary structure and ASA were obtained with the program, DSSP [20].

#### Experimental methods and conditions

Buffer name, buffer concentration, ion name, ion concentration, additives, protein concentration, measure and experimental method

#### Functional data

Aggregation rate, and change in aggregation rate (β aggregation rate) along with the parameters used in the experiment to account protein aggregation such as aggregation rate, apparent growth rate and relative fluorescence intensity.

#### Literature information

Keywords, reference, authors, PMID, year and remarks.

### Aggregating Peptides (AP)

This class contains the data on experimentally known amyloid fibril forming peptides, amorphous β-aggregating peptides, amyloid fibril forming peptides of different lengths, amyloid fibril forming hexa-peptides with three-dimensional structures available in the Protein Data Bank.

The four categories in this class are:

#### Amyloid fibril forming hexa-peptides

Contain 139 data obtained from our recent computational analysis [14], 244 data from WALTZ-DB [18] and a non-redundant set of 285 hexa-peptides from the combined dataset, obtained by discarding peptides with identical sequences.

#### Amyloid forming peptides of different lengths

Contain the aggregating peptides of lengths 7 to 72 residues. It also includes a few short aggregating peptides of five residues.

#### Amorphous β-aggregating hexapeptides and non-amyloids

Include a set of 168 amorphous peptides used in GAP [14] and 845 non-amyloids listed in WALTZ-DB [18]. Note that non-amyloids in WALTZ-DB refer to the peptides that do not form Amyloid-fibrils. Information on whether these peptides remain monomers or form amorphous β-aggregates is presently not available.

#### Amyloid fibril forming peptides with crystal structures

It has a set of 15 hexa-peptides of known structures along with their PDB codes [10].

### Aggregation Prone Regions (APR)

Amyloidogenic proteins contain one or more aggregating peptides known as aggregation prone regions, which nucleate protein aggregation. This dataset includes the name of the protein, amino acid sequence and aggregation prone regions. We have provided options to search with protein name or peptide sequence.

## Database Statistics

The first release of CPAD contains a total of about 4100 data on different categories. Specifically, 2356 data on change in aggregation rates upon mutations, including 1658 single point mutations and 586 wild-type data, 286 amyloid fibril forming hexa-peptides, 168 amorphous β-aggregating hexapeptides, 845 WALTZdb non-amyloid hexapeptides, 23 amyloid fibril forming hexa-peptides whose crystal structures are available in the PDB [10], 359 β-strand forming hexa-peptides from globular proteins, 76 experimentally validated APRs which include 47 APRs, with non-identical sequences, found in 33 amyloidogenic proteins.

## Features of CPAD

**Fig 1:**
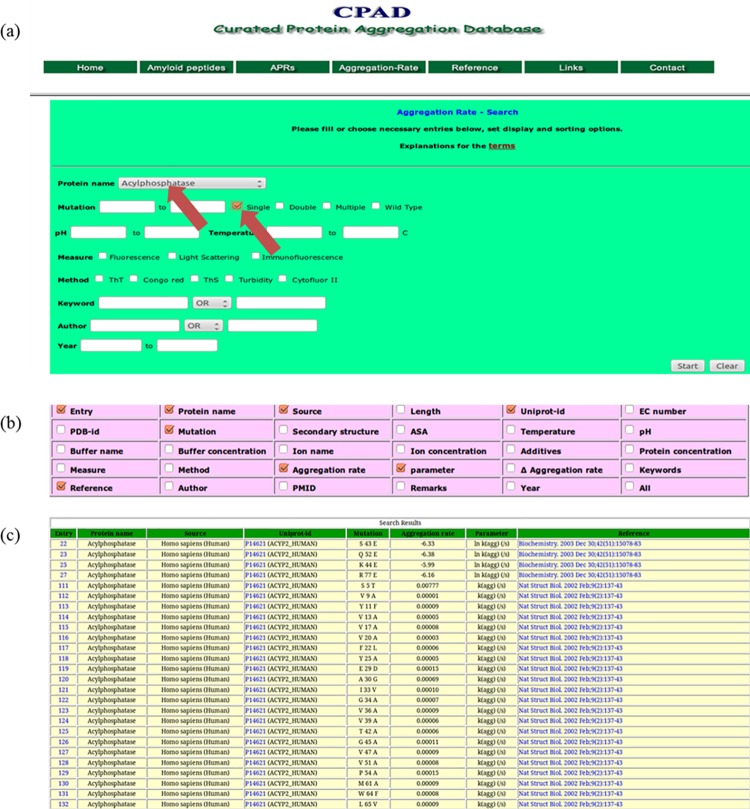
An example of searching conditions, display options and results of CPAD: (a) main menu for the search options in CPAD. The single mutants in Acylphosphatase are selected for search as indicated by arrows; (b) display options in CPAD. We have selected entry, protein name, source, Uniprot code, mutation, aggregation rate, parameter and reference to show in the output; (c) part of the results obtained from CPAD.

The features in CPAD are classified into two categories. In the first category, we provide the data on change in aggregation rates upon mutations, which include the features mentioned in the search and display options shown in **Fig 1** and are listed below:

1. Retrieve data for a particular protein
2. Type of mutant such as single, double, multiple and/or wild-type.
3. Experimental conditions such as pH range and temperature
4. Experimental methods such as Congo red, Thioflavin T, turbidity etc.
5. Extract information by Author names, publication year and keywords
6. Download the entire database

Detailed tutorials describing the usage of CPAD for retrieving the data on change in aggregation rate upon mutations are available at the home page of CPAD. For example, the data obtained with single mutants in Acylphosphatase is shown in **Fig 1A**. The terms entry, protein name, source, Uniprot code, mutation, aggregation rate, parameter and reference have been selected for displaying the results (**Fig 1B**). **Fig 1C** shows the final results obtained with the search conditions and display options.

In the second category, we provide the data on peptide sequences and APRs in amyloidogenic proteins. These data can be directly downloaded from the website of CPAD. Further, we have provided the details about the frequency of occurrence of amino acid residues at different positions of amyloid forming, amorphous and non-amyloid hexapeptides and their average hydrophobicity values. The average contacts between amino acid residues for the aggregating peptides of known structures are also given along with the list of peptides.

## Potential Uses of CPAD

CPAD contains aggregation data from different perspectives. A few broad potential applications of CPAD are described below: (i) the data on aggregation rates upon mutations can be used to understand factors influencing protein aggregation and help develop computational models for distinguishing between the mutants, which increase or decrease aggregation, as well as for predicting the aggregations rates upon mutation(s). Such tools can contribute to design of protein/peptide aggregate containing nanomaterials with desirable properties, for stabilizing industrial enzymes and for finding novel cures for neurodegenerative and other aggregation mediated diseases [21–25]. (ii) The experimental data on aggregation prone regions (APRs) can be used to develop and validate algorithms capable of predicting such regions in amino acid sequences and study their sequence–structural overlap/ adjacency with the regions which code for, say, protein: protein interfaces, immune epitopes, catalytic sites, disordered regions, etc. [26,27]. (iii) Collection of sequence regions underpinning different aggregate morphologies, amyloid-fibril or amorphous-β, can also be very useful in understanding the risk of formation of different types of aggregates in different biotechnological products because they can lead to improved strategies for mitigating aggregation. On the other hand, knowledge of such sequences is critical to design of novel aggregates with well-defined morphologies for nanotechnology applications. At the level of fundamental research, it is important to decipher why certain aggregation prone regions code for amyloid-fibrils, but not amorphous-β aggregates and vice versa. (iv) The various types of experimental data collected in CPAD can also be used to perform comparative proteome wide studies on risk of aggregation to different organisms living in their environment. Such studies shall help us understand how organisms living under extreme environments deal with aggregation [16]. (v) CPAD also lists a set of web servers for identifying aggregating peptides and APRs that can prove useful for benchmarking studies across different methods of aggregation prediction. The users of CPAD are encouraged to apply the CPAD collections in innovative ways. In summary, it is envisaged that CPAD will serve as a very useful resource to scientific community interested in understanding different facets of protein aggregation such as its role in protein folding and stability, protein evolution and adaptation, finding novel cures to age-related diseases and for engineering novel nanomaterials.

## Links to Other Databases

Each entry in CPAD is linked to Uniprot ID (http://www.uniprot.org/) and PDB code (http://www.rcsb.org) so that the users can obtain the sequence and structure information directly. The references for all data are directly connected to the PUBMED literature database (http://www.ncbi.nlm.nih.gov/pubmed/). Further, we have provided links to several related databases and web servers on protein aggregation.

## Availability and Citation of CPAD

The database can be freely accessible at http://www.iitm.ac.in/bioinfo/CPAD/. If this database is used as a tool in your published research work, please cite this article including the URL. Suggestions and comments are welcome and should be sent to gromiha@iitm.ac.in.

## Submission of Data to CPAD

We have provided a facility for submitting the data on aggregating, amorphous and non-amyloid peptides, aggregation prone regions in amyloidogenic proteins and aggregation rates to CPAD by the authors. The data will be reviewed and uploaded in the database. We encourage the authors to provide their experimental data either upload at the website or send to gromiha@iitm.ac.in.
